# Different impacts of granulocyte colony‐stimulating factor administration on allogeneic hematopoietic cell transplant outcomes for adult acute myeloid leukemia according to graft type

**DOI:** 10.1002/ajh.27521

**Published:** 2024-11-20

**Authors:** Takaaki Konuma, Kazuaki Kameda, Kaoru Morita, Tadakazu Kondo, Fumihiko Kimura, Hideki Nakasone, Fumihiko Ouchi, Naoyuki Uchida, Masatsugu Tanaka, Tetsuya Nishida, Takahiro Fukuda, Yuta Hasegawa, Mamiko Sakata‐Yanagimoto, Makoto Onizuka, Masashi Sawa, Shuichi Ota, Noboru Asada, Shin‐Ichiro Fujiwara, Satoshi Yoshihara, Fumihiko Ishimaru, Makoto Yoshimitsu, Yoshinobu Kanda, Marie Ohbiki, Yoshiko Atsuta, Masamitsu Yanada

**Affiliations:** ^1^ Department of Hematology/Oncology, The Institute of Medical Science The University of Tokyo Tokyo Japan; ^2^ Division of Hematology Jichi Medical University Saitama Medical Center Saitama Japan; ^3^ Division of Hematology Jichi Medical University Shimotsuke Japan; ^4^ Department of Hematology Kobe City Medical Center General Hospital Kobe Japan; ^5^ Division of Hematology, Department of Internal Medicine National Defense Medical College Tokorozawa Japan; ^6^ Division of Stem Cell Regulation, Center for Molecular Medicine Jichi Medical University Shimotsuke Japan; ^7^ Hematology Division Tokyo Metropolitan Cancer and Infectious Diseases Center, Komagome Hospital Tokyo Japan; ^8^ Department of Hematology Toranomon Hospital Tokyo Japan; ^9^ Department of Hematology Kanagawa Cancer Center Yokohama Japan; ^10^ Department of Hematology Japanese Red Cross Aichi Medical Center Nagoya Daiichi Hospital Nagoya Japan; ^11^ Department of Hematopoietic Stem Cell Transplantation National Cancer Center Hospital Tokyo Japan; ^12^ Department of Hematology Hokkaido University Hospital Sapporo Japan; ^13^ Department of Hematology University of Tsukuba Hospital Tsukuba Japan; ^14^ Department of Hematology and Oncology Tokai University School of Medicine Isehara Japan; ^15^ Department of Hematology and Oncology Anjo Kosei Hospital Anjo Japan; ^16^ Department of Hematology Sapporo Hokuyu Hospital Sapporo Japan; ^17^ Department of Hematology and Oncology Okayama University Hospital Okayama Japan; ^18^ Department of Hematology Hyogo Medical University Hospital Nishinomiya Japan; ^19^ Technical Department Japanese Red Cross Society Blood Service Headquarters Tokyo Japan; ^20^ Department of Hematology and Rheumatology Kagoshima University Hospital Kagoshima Japan; ^21^ Japanese Data Center for Hematopoietic Cell Transplantation Nagakute Japan; ^22^ Department of Registry Science for Transplant and Cellular Therapy Aichi Medical University School of Medicine Nagakute Japan; ^23^ Department of Hematology and Oncology Nagoya University Graduate School of Medicine Nagoya Japan; ^24^ Department of Hematology and Oncology Nagoya City University East Medical Center Nagoya Japan

## Abstract

We retrospectively evaluated the impacts of using granulocyte colony‐stimulating factor (G‐CSF) and its timing on posttransplant outcomes for 9766 adults with acute myeloid leukemia (AML) between 2013 and 2022 using a Japanese database. We separately evaluated three distinct cohorts based on graft type: 3248 received bone marrow transplantation (BMT), 3066 received peripheral blood stem cell transplantation (PBSCT), and 3452 received single‐unit cord blood transplantation (CBT). Multivariate analysis showed that G‐CSF administration significantly accelerated neutrophil recovery after BMT, PBSCT, and CBT. However, it was associated with a higher risk of grades II–IV acute graft‐versus‐host disease (GVHD) across all graft types. Moreover, an increased incidence of overall chronic GVHD was observed with G‐CSF administration in BMT and CBT patients, but not in PBSCT patients. G‐CSF administration significantly improved overall survival (OS) and leukemia‐free survival (LFS) only following CBT. Regarding the timing of G‐CSF, in comparison with late initiation of G‐CSF (Days 5–10), early initiation (Days 0–4) did not provide benefits for hematopoietic recovery regardless of graft type. In contrast, late initiation was significantly associated with a lower risk of grades II–IV acute GVHD and better OS and LFS in CBT patients. These data demonstrated that G‐CSF administration accelerated neutrophil recovery and increased the risk of grades II–IV acute GVHD across all graft types, but significantly improved survival outcomes but only following CBT. Therefore, routine use of G‐CSF should be considered for CBT in adult patients with AML.

## 1 INTRODUCTION

Granulocyte colony‐stimulating factor (G‐CSF) enhances myeloid progenitor proliferation and differentiation. G‐CSF has been widely used to accelerate neutrophil recovery after allogeneic hematopoietic cell transplantation (HCT) at many transplant centers. Administration of G‐CSF has been shown to shorten the time to neutrophil engraftment,^1^, ^2^, ^3^, ^4^, ^5^, ^6^, ^7^, ^8^, ^9^, ^10^, ^11^, ^12^, ^13^ reduce febrile episodes,^3^ and shorten hospital stays^4^, ^10^, ^11^, ^13^ compared to no administration of G‐CSF following allogeneic HCT. However, G‐CSF administration has also been associated with an increased incidence of acute and chronic graft‐versus‐host disease (GVHD)^6^, ^7^, ^9^, ^13^ and delayed platelet recovery.^7^, ^9^, ^12^ Moreover, due to its potential to induce acute myeloid leukemia (AML) cell proliferation,^14^ concerns about the increased risk of relapse in myeloid leukemia, especially with early G‐CSF initiation following HCT, have been raised. Recent advancements in allogeneic HCT for AML, including extended graft availability, conditioning regimens, and GVHD prophylaxis, may improve posttransplant outcomes.^15^, ^16^, ^17^ Although the time to hematopoietic recovery and incidence of acute and chronic GVHD are influenced by graft type,^18^, ^19^ the optimal use of G‐CSF and timing of its initiation following allogeneic HCT for AML remain unclear. In this study, we performed a retrospective analysis using a nationwide Japanese database of a large cohort of adult AML patients treated with allogeneic bone marrow transplantation (BMT), peripheral blood stem cell transplantation (PBSCT), or single‐unit cord blood transplantation (CBT) to evaluate the impact of G‐CSF use and timing on posttransplant outcomes.

## 2 METHODS

### 2.1 Data collection

This retrospective study was conducted by the Adult AML and the Donor/Source Working Group of the Japanese Society for Transplantation and Cellular Therapy (JSTCT). Clinical data were extracted from the Transplant Registry Unified Management Program of the Japanese Data Center for Hematopoietic Cell Transplantation (JDCHCT) and the JSTCT.^20^, ^21^ The study included patients aged between 16 and 65 years who underwent their first allogeneic BMT, PBSCT, or single‐unit CBT between 2013 and 2022 in Japan, because the upper age limit for allogeneic HCT is 65 years old in several transplant centers during the study periods. Patients who did not receive G‐CSF or received G‐CSF after graft infusion were eligible for this study if the first G‐CSF administration occurred between Days 0 and 10, G‐CSF was initiated before neutrophil engraftment, and the duration of administration of G‐CSF exceeded 3 days. Ultimately, 9766 patients who met these criteria were enrolled. This study was approved by the adult AML and the Donor/Source Working Group of the JSTCT and by the institutional review board of the Institute of Medical Science, The University of Tokyo (2023‐56‐1019), where the study was conducted.

### 2.2 Study objectives

The primary objective was to investigate the impact of G‐CSF administration on acute and chronic GVHD for each graft type. The secondary objective was to investigate the impact of G‐CSF administration on neutrophil and platelet recovery, relapse, non‐relapse mortality (NRM), overall survival (OS), and leukemia‐free survival (LFS) for each graft type.

### 2.3 Definitions

The diagnosis and severity of GVHD were based on previously established standard criteria.^22^, ^23^ Neutrophil recovery was defined as an absolute neutrophil count exceeding 0.5 × 10^9^/L on 3 consecutive days. Platelet recovery was characterized by a platelet count exceeding 20 × 10^9^/L on 7 consecutive days following the last platelet transfusion. Relapse was defined as morphologic evidence of AML. NRM was defined as death during remission. OS (the inverse of overall mortality) was defined as the time from HCT to death from any cause, and LFS was defined as the time from HCT to relapse or death from any cause. Surviving patients were censored at the time of the last observation. The hematopoietic cell transplantation‐specific comorbidity index (HCT‐CI),^24^ Eastern Cooperative Oncology Group performance status (PS),^25^ cytogenetic risk,^26^ and intensity of conditioning regimen^27^ were classified according to published criteria. The number of human leukocyte antigen (HLA) CSP/TAC disparities between recipient and donor was defined based on low‐resolution HLA‐A, ‐B, and ‐DR matching in the graft‐versus‐host direction. Complete remission (CR) was determined by the presence of less than 5% bone marrow blasts.

### 2.4 Statistical analysis

Group comparisons were conducted using chi‐squared or Fisher's exact tests for categorical variables and Kruskal–Wallis tests for continuous variables. Cumulative incidence estimates were used to calculate the unadjusted cumulative incidence of GVHD, hematopoietic recovery, relapse, and NRM, which were compared using Gray's test. The Kaplan–Meier method was used to estimate the unadjusted probability of OS and LFS, which were compared using the log‐rank test. Multivariate analyses used the Fine and Gray proportional hazards model for GVHD, hematopoietic recovery, relapse, and NRM, and the Cox proportional hazards regression model for overall mortality (1‐OS) and treatment failure (1‐LFS). Hazard ratios (HR) with 95% confidence intervals (CI) were estimated for G‐CSF administration (no administration vs. administration), or administration and timing of G‐CSF initiation (no administration vs. 0–4 vs. 5–10 days), adjusting for covariates: age (<50 vs. ≥50 years), recipient sex (male vs. female), PS (0–1 vs. 2–4), HCT‐CI (0–2 vs. ≥3), cytogenetic risk (other than poor vs. poor), disease status at HCT (CR vs. non‐CR), conditioning regimen (myeloablative conditioning [MAC] vs. reduced‐intensity conditioning [RIC]), GVHD prophylaxis (calcineurin inhibitors and methotrexate vs. others), use of antithymocyte globulin (ATG) (without ATG vs. with ATG), HLA disparities (match vs. mismatch), and year of HCT (2013–2017 vs. 2018–2022). For BMT and PBSCT, donor type (related vs. unrelated) was also included as a variable in the multivariate analysis. Analyses were conducted separately for each cohort based on graft type (BMT, PBSCT, and CBT).

Statistical analyses were performed using EZR version 1.68 (Saitama Medical Center, Jichi Medical University),^28^ a graphical user interface for R 4.4.0 software (R Foundation for Statistical Computing). Two‐sided *p*‐values are reported, with *p* < .05 considered significant.

## 3 RESULTS

### 3.1 Characteristics of patients and transplant procedures

The characteristics of patients and transplant procedures are summarized in Table 1. Among 9766 patients, 3248 received BMT, 3066 received PBSCT, and 3452 received CBT. The median recipient age at HCT was 51 years for BMT, 49 years for PBSCT, and 52 years for CBT recipients (*p* < .001). BMT recipients were less likely to have PS 2–4 (*p* < .001) and HCT‐CI ≥ 3 (*p* < .001). CBT recipients were more likely to have poor cytogenetic risk (*p* < .001) and non‐CR status (*p* < .001). A higher proportion of PBSCT recipients received the RIC regimen (*p* < .001), whereas calcineurin inhibitors and methotrexate‐based GVHD prophylaxis regimens were predominantly used in BMT recipients (*p* < .001). PBSCT recipients were more likely to receive ATG (*p* < .001). Differences were observed among graft types in donor type (*p* < .001), numbers of HLA disparities (*p* < .001), and year of HCT (*p* < .001). The proportion of G‐CSF administration was 79.8% in BMT, 81.5% in PBSCT, and 87.8% in CBT recipients (*p* < .001).

**TABLE 1:** Characteristics of patient, disease, and transplantation according to graft source.

	BMT	PBSCT	CBT	*p*
Number of patients	3248	3066	3452	
Median recipient age, years (IQR)	51 (41–59)	49 (38–57)	52 (42–60)	**<.001**
Recipient age, number (%)				**<.001**
<50 years	1502 (46.2)	1582 (51.6)	1470 (42.6)	
≥50 years	1746 (53.8)	1484 (48.4)	1982 (57.4)	
Recipient sex, number (%)				**.003**
Male	1394 (42.9)	1222 (39.9)	1513 (43.8)	
Female	1853 (57.1)	1844 (60.1)	1938 (56.2)	
Missing	1	0	1	
Performance status, number (%)				**<.001**
0–1	3038 (93.6)	2785 (90.9)	3132 (90.8)	
2–4	207 (6.4)	279 (9.1)	318 (9.2)	
Missing	3	2	2	
HCT‐CI, number (%)				**<.001**
0–2	2761 (85.4)	2496 (82.1)	2805 (81.9)	
≥3	473 (14.6)	543 (17.9)	619 (18.1)	
Missing	14	27	28	
Cytogenetic risk, number (%)				**<.001**
Favorable	397 (12.2)	366 (11.9)	359 (10.4)	
Intermediate	1979 (60.9)	1802 (58.8)	1987 (57.6)	
Poor	648 (20.0)	726 (23.7)	921 (26.7)	
Unevaluable	224 (6.9)	172 (5.6)	185 (5.4)	
Disease status, number (%)				**<.001**
CR	2270 (71.0)	1810 (59.9)	1821 (53.3)	
Non‐CR	927 (29.0)	1211 (40.1)	1593 (46.7)	
Missing	51	45	38	
Conditioning regimen, number (%)				**<.001**
MAC	2524 (77.7)	2145 (70.0)	2577 (74.7)	
RIC	724 (22.3)	921 (30.0)	875 (25.3)	
GVHD prophylaxis, number (%)				**<.001**
CSP/TAC + MTX	3018 (93.2)	1964 (64.2)	1824 (53.0)	
CSP/TAC + MMF	110 (3.4)	776 (25.4)	1363 (39.6)	
Others	109 (3.4)	317 (10.4)	256 (7.4)	
Missing	11	9	9	
ATG, number (%)				**<.001**
No use	2898 (89.2)	2435 (79.4)	3379 (97.9)	
Use of ATG	350 (10.8)	631 (20.6)	73 (2.1)	
PTCy, number (%)				**<.001**
No PTCy	1791 (98.2)	1559 (70.6)	2396 (99.9)	
Use of PTCy	33 (1.8)	648 (29.4)	3 (0.1)	
Donor type, number (%)				**<.001**
Related	507 (15.6)	2575 (84.0)	0 (0.0)	
Unrelated	2741 (84.4)	491 (16.0)	3452 (100.0)	
HLA mismatch, number (%)				**<.001**
0	2608 (80.3)	1850 (60.4)	257 (7.5)	
1	597 (18.4)	364 (11.9)	1154 (33.5)	
≥2	41 (1.3)	848 (27.7)	2038 (59.1)	
Missing	2	4	3	
HCT year, number (%)				**<.001**
2013–2017	1906 (58.7)	1359 (44.3)	1636 (47.4)	
2018–2022	1342 (41.3)	1707 (55.7)	1816 (52.6)	
G‐CSF, number (%)				**<.001**
No administration	657 (20.2)	568 (18.5)	422 (12.2)	
Administration	2591 (79.8)	2498 (81.5)	3030 (87.8)	

*Note*: The *p*‐values in bold are statistically significant (<.05).

Abbreviations: ATG, antithymocyte globulin; BMT, bone marrow transplantation; CBT, cord blood transplantation; CR, complete remission; CSP, cyclosporine; G‐CSF, granulocyte colony‐stimulating factor; GVHD, graft‐versus‐host disease; HCT, hematopoietic cell transplantation; HCT‐CI, hematopoietic cell transplantation‐specific comorbidity index; IQR, interquartile range; MAC, myeloablative conditioning; MMF, mycophenolate mofetil; MTX, methotrexate; PBSCT, peripheral blood stem cell transplantation; PTCy, posttransplant cyclophosphamide; RIC, reduced‐intensity conditioning, TAC, tacrolimus.

### 3.2 Impact of G‐CSF administration on transplant outcomes

**FIGURE 1:**
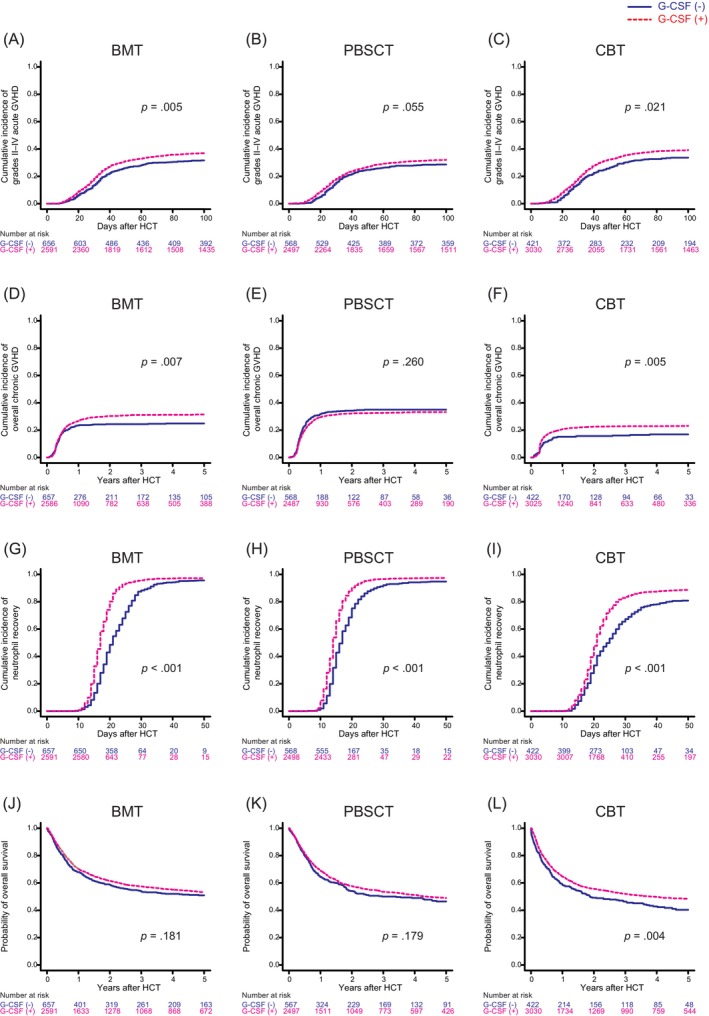
Effect of granulocyte colony‐stimulating factor (G‐CSF) administration on grades II–IV acute graft‐versus‐host disease (GVHD), overall chronic GVHD, neutrophil recovery, and overall survival according to graft type. BMT, bone marrow transplantation; CBT, cord blood transplantation; HCT, hematopoietic cell transplantation; PBSCT, peripheral blood stem cell transplantation. [Color figure can be viewed at wileyonlinelibrary.com]

In the univariate analysis, the cumulative incidence of grades II–IV acute GVHD was significantly higher in patients receiving G‐CSF compared to those not receiving it, regardless of graft type (*p* = .005 for BMT, *p* = .055 for PBSCT, *p* = .021 for CBT) (Figure 1A–C). In the multivariate analysis, administration of G‐CSF was significantly associated with a higher risk of grades II–IV acute GVHD across all graft types (HR 1.24, 95% CI 1.06–1.44, *p* = .005 for BMT; HR 1.27, 95% CI 1.07–1.52, *p* = .006 for PBSCT; HR 1.21, 95% CI 1.01–1.43, *p* = .030 for CBT) (Table S1). There was no significant difference in grades III and IV acute GVHD between patients receiving G‐CSF and those not receiving it in both the univariate and multivariate analyses (Figure S1A–C); Table S1).

In the univariate analysis, the cumulative incidence of overall chronic GVHD was significantly higher in patients receiving G‐CSF compared to those not receiving it following BMT (*p* = .007) and CBT (*p* = .005), but not PBSCT (*p* = .260) (Figure 1D–F). In the multivariate analysis, administration of G‐CSF was significantly associated with a higher risk of overall chronic GVHD following BMT (HR 1.20, 95% CI 1.00–1.43, *p* = .040) and CBT (HR 1.42, 95% CI 1.10–1.84, *p* = .007) (Table S1). In the univariate analysis, the cumulative incidence of extensive chronic GVHD was significantly higher in patients receiving G‐CSF compared to those not receiving it following BMT (*p* < .001), but not PBSCT (*p* = .951) or CBT (*p* = .102) (Figure S1D–F). In the multivariate analysis, administration of G‐CSF was significantly associated with a higher risk of extensive chronic GVHD following BMT (HR 1.46, 95% CI 1.13–1.87, *p* = .002) (Table S1).

In the univariate analysis, the cumulative incidence of neutrophil recovery was significantly accelerated in patients receiving G‐CSF compared to those not receiving it, irrespective of graft type (*p* < .001 for BMT, *p* < .001 for PBSCT, and *p* < .001 for CBT) (Figure 1G–I). In the multivariate analysis, administration of G‐CSF was significantly associated with an accelerated neutrophil recovery, regardless of graft type (HR 1.77, 95% CI 1.64–1.91, *p* < .001 for BMT; HR 1.63, 95% CI 1.50–1.77, *p* < .001 for PBSCT; HR 1.44, 95% CI 1.30–1.60, *p* < .001 for CBT) (Table S1).

In the univariate analysis, the cumulative incidence of platelet recovery was significantly slower in patients receiving G‐CSF compared to those not receiving it following PBSCT (*p* < .001), but not BMT (*p* = .435) or CBT (*p* = .419) (Figure S1G–I). In the multivariate analysis, administration of G‐CSF was significantly associated with a slower platelet recovery following BMT (HR 0.89, 95% CI 0.80–0.98, *p* = .019) and PBSCT (HR 0.83, 95% CI 0.74–0.92, *p* = .001) (Table S1).

In the univariate analysis, the cumulative incidence of relapse was significantly lower in patients receiving G‐CSF compared to those not receiving it following PBSCT (*p* = .043) or CBT (*p* = .026), but not BMT (*p* = .342) (Figure S2A–C). In the multivariate analysis, there was no significant difference in relapse between patients receiving G‐CSF and those not receiving it for each donor type (Table S1). In both the univariate and multivariate analyses, there was no significant difference in NRM between patients receiving G‐CSF and those not receiving it for each donor type (Figure S2D–F; Table S1).

In the univariate analysis, the probability of OS was significantly higher in patients receiving G‐CSF compared to those not receiving it following CBT (*p* = .004), but not BMT (*p* = .181) or PBSCT (*p* = .179) (Figure 1J–L). In the multivariate analysis, administration of G‐CSF was significantly associated with better OS following CBT (HR 0.79, 95% CI 0.68–0.91, *p* = .001) (Table S1). In the univariate analysis, the probability of LFS was significantly higher in patients receiving G‐CSF compared to those not receiving it following PBSCT (*p* = .021) or CBT (*p* = .022), but not BMT (*p* = .378) (Figure S2G–I). In the multivariate analysis, administration of G‐CSF was significantly associated with better LFS following CBT (HR 0.82, 95% CI 0.71–0.95, *p* = .008) (Table S1).

### 3.3 Subgroup impact of G‐CSF administration on transplant outcomes

**FIGURE 2:**
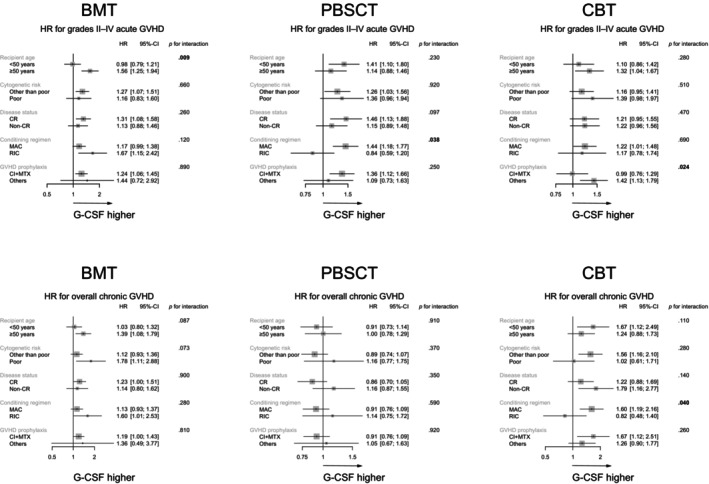
Forest plots showing adjusted hazard ratios and 95% confidence intervals (CI) of granulocyte colony‐stimulating factor (G‐CSF) administration for grades II–IV acute graft‐versus‐host disease (GVHD) and overall chronic GVHD among each graft type in the subgroup analysis. BMT, bone marrow transplantation; CBT, cord blood transplantation; CI, confidence interval; CI, calcineurin inhibitor; CR, complete remission; HCT, hematopoietic cell transplantation; HR, hazard ratios; MAC, myeloablative conditioning; MTX, methotrexate; PBSCT, peripheral blood stem cell transplantation; RIC, reduced‐intensity conditioning.

We also evaluated the impact of G‐CSF administration on grades II–IV acute GVHD, overall chronic GVHD, neutrophil recovery, relapse, and OS stratified by recipient age, cytogenetic risk, disease status, conditioning regimen, and GVHD prophylaxis for each donor type. The effect of G‐CSF administration on grades II–IV acute GVHD was significant based on recipient age following BMT (*p* for interaction = .009), conditioning regimen following PBSCT (*p* for interaction = .038), and GVHD prophylaxis following CBT (*p* for interaction = .024). Similarly, the effect of G‐CSF administration on overall chronic GVHD was significant based on the conditioning regimen following CBT (*p* for interaction = .040) (Figure 2). Administration of G‐CSF did not increase the risk of AML relapse following BMT, PBSCT, or CBT, regardless of cytogenetic risk or disease status at HCT, except for a significant effect of G‐CSF administration on relapse based on disease status following PBSCT (*p* for interaction = .049) (Figure S3).

The effect of G‐CSF administration on neutrophil recovery was significant based on GVHD prophylaxis following PBSCT (*p* for interaction = .019), and on disease status (*p* for interaction<.001) and GVHD prophylaxis (*p* for interaction = .001) following CBT. Furthermore, the effect of G‐CSF administration on overall mortality was significant based on the conditioning regimen following PBSCT (*p* for interaction = .029) and the conditioning regimen following CBT (*p* for interaction = .025) (Figure S3).

### 3.4 Impact of timing of G‐CSF initiation on transplant outcomes

We also evaluated the impact of the timing of G‐CSF initiation on transplant outcomes according to graft type. The median initiation time of G‐CSF initiation after graft infusion was 5 days for BMT, PBSCT, and CBT. Hence, in univariate and multivariate analysis, we investigated whether administration and timing of G‐CSF initiation (no administration vs. 0–4 vs. 5–10 days) affect transplant outcomes.

**FIGURE 3:**
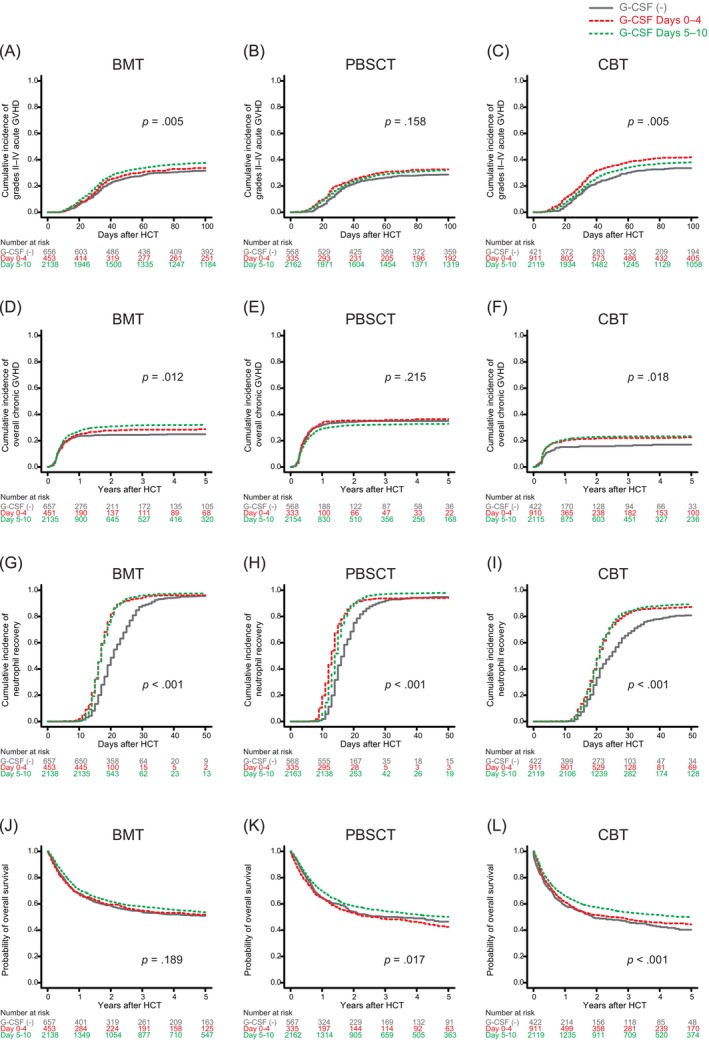
Effect of administration and timing of granulocyte colony‐stimulating factor (G‐CSF) initiation on grades II–IV acute graft‐versus‐host disease (GVHD), overall chronic GVHD, neutrophil recovery, and overall survival according to graft type. BMT, bone marrow transplantation; CBT, cord blood transplantation; HCT, hematopoietic cell transplantation; PBSCT, peripheral blood stem cell transplantation. [Color figure can be viewed at wileyonlinelibrary.com]

In the univariate analysis, the cumulative incidence of grades II–IV acute GVHD significantly varied with the administration and timing of G‐CSF initiation following BMT (*p* = .005) and CBT (*p* = .005), but not PBSCT (*p* = .158) (Figure 3A–C). In the multivariate analysis, early initiation of G‐CSF (Days 0–4) was significantly associated with a higher risk of grades II–IV acute GVHD following CBT compared to those not receiving it (HR 1.33, 95% CI 1.09–1.61, *p* = .003). Conversely, late initiation of G‐CSF (Days 5–10) was significantly associated with a higher risk of grades II–IV acute GVHD following BMT (HR 1.27, 95% CI 1.09–1.48, *p* = .002) and PBSCT (HR 1.28, 95% CI 1.07–1.53, *p* = .006) compared to those not receiving it. Compared to early initiation of G‐CSF, late initiation of G‐CSF was significantly associated with a lower risk of grades II–IV acute GVHD following CBT (HR 0.87, 95% CI 0.76–0.98, *p* = .032) (Table S2). There was no significant difference in grades III and IV acute GVHD among different timings of G‐CSF initiation for each donor type in both the univariate and multivariate analyses (Figure S4A–C; Table S2).

In the univariate analysis, the cumulative incidence of overall chronic GVHD significantly differed with the administration and timing of G‐CSF initiation following BMT (*p* = .012) and CBT (*p* = .018), but not PBSCT (*p* = .215) (Figure 3D–F). In the multivariate analysis, early initiation of G‐CSF was significantly associated with a higher risk of overall chronic GVHD following CBT (HR 1.37, 95% CI 1.03–1.83, *p* = .028) compared to those not receiving it. Late initiation of G‐CSF was significantly associated with a higher risk of overall chronic GVHD following BMT (HR 1.22, 95% CI 1.02–1.47, *p* = .025) and CBT (HR 1.44, 95% CI 1.11–1.87, *p* = .006) compared to those not receiving it (Table S2).

In the univariate analysis, the cumulative incidence of extensive chronic GVHD significantly varied with the administration and timing of G‐CSF initiation following BMT (*p* = .002) (Figure S4D–F). In the multivariate analysis, late initiation of G‐CSF was significantly associated with a higher risk of extensive chronic GVHD following BMT (HR 1.48, 95% CI 1.15–1.91, *p* = .002) compared to those not receiving it (Table S2).

In the univariate analysis, the cumulative incidence of neutrophil recovery significantly varied with the administration and timing of G‐CSF initiation, irrespective of graft type (*p* < .001 for BMT, *p* < .001 for PBSCT, *p* < .001 for CBT) (Figure 3G–I). In the multivariate analysis, early and late initiation of G‐CSF was significantly associated with an accelerated neutrophil recovery across all graft types (Table S2). In the univariate analysis, the cumulative incidence of platelet recovery varied with the administration and timing of G‐CSF initiation following PBSCT (*p* = .001), but not BMT (*p* = .583) or CBT (*p* = .669) (Figure S4G–I). In the multivariate analysis, late initiation of G‐CSF was significantly associated with a slower platelet recovery following BMT (HR 0.88, 95% CI 0.80–0.97, *p* = .013) and PBSCT (HR 0.81, 95% CI 0.73–0.91, *p* < .001) compared to those not receiving it (Table S2).

In the univariate analysis, the cumulative incidence of relapse did not differ with the administration and timing of G‐CSF initiation for any graft type (*p* = .631 for BMT, *p* = .064 for PBSCT, *p* = .060 for CBT) (Figure S5A–C). In the multivariate analysis, early initiation of G‐CSF was significantly associated with a lower risk of relapse following PBSCT (HR 0.69, 95% CI 0.53–0.89, *p* = .005) compared to those not receiving it, whereas late initiation of G‐CSF was significantly associated with a higher risk of relapse following PBSCT (HR 1.32, 95% CI 1.05–1.66, *p* = .014) compared to early initiation of it (Table S2).

In the univariate analysis, the cumulative incidence of NRM significantly differed with the administration and timing of G‐CSF initiation following PBSCT (*p* = .002), but not BMT (*p* = .642) or CBT (*p* = .129) (Figure S5D–F). In the multivariate analysis, late initiation of G‐CSF was significantly associated with a lower risk of NRM following PBSCT (HR 0.67, 95% CI 0.52–0.86, *p* = .002) compared to early initiation of it (Table S2).

In the univariate analysis, the probability of OS significantly varied with the administration and timing of G‐CSF initiation following PBSCT (*p* = .017) or CBT (*p* < .001), but not BMT (*p* = .189) (Figure 3J–L). In the multivariate analysis, late initiation of G‐CSF was significantly associated with better OS following CBT compared to those not receiving it (HR 0.75, 95% CI 0.65–0.88, *p* < .001) and compared to early initiation of it (HR 0.87, 95% CI 0.78–0.97, *p* = .019) (Table S2).

In the univariate analysis, the probability of LFS significantly varied with the administration and timing of G‐CSF initiation following PBSCT (*p* = .020) or CBT (*p* = .002), but not BMT (*p* = .474) (Figure S5G–I). In the multivariate analysis, late initiation of G‐CSF was significantly associated with better LFS following CBT compared to those not receiving it (HR 0.79, 95% CI 0.68–0.91, *p* = .001) and early initiation of it (HR 0.86, 95% CI 0.77–0.96, *p* = .012) (Table S2).

We also evaluated whether administration and timing of G‐CSF initiation (no administration vs. 0–4 vs. 5–10 days) affects time‐bound NRM. In the univariate analysis, the cumulative incidence of 6 months NRM significantly differed with the administration and timing of G‐CSF initiation following BMT (*p* = .011) and PBSCT (*p* < .001), but not CBT (*p* = .339) (Figure S6A–C). In the multivariate analysis, early initiation of G‐CSF was significantly associated with a higher risk of NRM following BMT and PBSCT compared to those not receiving it and early initiation of it (Table S3). In the univariate analysis, the cumulative incidence of 1‐year NRM significantly differed with the administration and timing of G‐CSF initiation following PBSCT (*p* = .005), but not BMT (*p* = .163) or CBT (*p* = .143) (Figure S6D–F). In the multivariate analysis, early initiation of G‐CSF was significantly associated with a higher risk of NRM following PBSCT compared to those not receiving it and early initiation of it (Table S3).

### 3.5 Impact of G‐CSF administration and timing of G‐CSF on haploidentical transplant outcomes

Finally, we evaluated the impact of G‐CSF administration and timing on outcomes after haploidentical transplantation, where donors were related and mismatched at two to three HLA antigen levels (HLA‐A, HLA‐B, and HLA‐DR) in the graft‐versus‐host direction. Among 871 recipients of haploidentical transplantation, peripheral blood stem cell grafts were more common (*n* = 831, 95.4%). Posttransplant cyclophosphamide (PTCy) was used for GVHD prophylaxis in 508 patients, whereas 255 patients received ATG. In the univariate analysis, the cumulative incidences of grades III and IV acute GVHD and neutrophil recovery, as well as the probabilities of OS and LFS, significantly varied based on the administration and timing of G‐CSF initiation. Early initiation of G‐CSF was associated with higher rates of grades III and IV acute GVHD and an accelerated neutrophil recovery. Additionally, late initiation of G‐CSF was associated with better OS and LFS (Figure S7).

## 4 DISCUSSION

Our study demonstrated that administration of G‐CSF led to an improvement in neutrophil recovery but was significantly associated with a higher risk of grades II–IV acute GVHD compared to no administration of G‐CSF, irrespective of graft type. Interestingly, administration of G‐CSF significantly improved OS and LFS only following CBT. Regarding the timing to initiate G‐CSF, compared with late initiation (Days 5–10), there was no benefit of early initiation (Days 0–4) for hematopoietic recovery regardless of graft type. In contrast, compared to early initiation, late initiation was significantly associated with a lower risk of grades II–IV acute GVHD and better OS and LFS in CBT recipients.

Although most previous studies did not show an association between G‐CSF administration and incidences of acute and chronic GVHD,^1^, ^2^, ^3^, ^4^, ^5^, ^8^, ^10^, ^12^ several studies demonstrated that administration of G‐CSF was associated with an increased risk of acute^6^, ^7^, ^9^ and chronic GVHD^7^, ^9^, ^13^ following BMT or PBSCT. A retrospective study from the European Group for Blood and Marrow Transplantation showed increased incidence of grades II–IV acute GVHD and chronic GVHD with G‐CSF administration among BMT patients, but not PBSCT patients.^7^ Our data also showed a significant increase in grades II–IV acute GVHD with G‐CSF administration following BMT, PBSCT, or CBT. Moreover, an increased incidence of overall chronic GVHD with G‐CSF administration was observed in BMT and CBT patients, but not PBSCT patients. This difference might be partly due to a higher frequency of haploidentical transplantation among PBSCT patients, which included frequent use of PTCy and ATG for GVHD prophylaxis. This could contribute to the lack of association between the incidence of overall chronic GVHD and G‐CSF administration in PBSCT patients. In addition, our data also showed a significant increase in grades II–IV acute GVHD with early initiation of G‐CSF following CBT. In contrast, late initiation of G‐CSF increased extensive chronic GVHD in BMT patients, but not CBT patients. These conflicting results suggest that the effect of early and late initiation of G‐CSF on GVHD needs further study.

Our data demonstrated that administration of G‐CSF significantly accelerated neutrophil recovery after allogeneic HCT, irrespective of graft type. In contrast, similar to previous studies,^7^, ^9^, ^12^ administration of G‐CSF significantly suppressed platelet recovery following BMT or PBSCT, but not CBT. This difference could be partly due to the delayed platelet recovery observed in CBT, which might counteract the negative impact of G‐CSF administration on platelet recovery. On the other hand, there was no significant difference in hematopoietic recovery between different timings of G‐CSF initiation after allogeneic HCT, consistent with several prospective studies.^29^, ^30^, ^31^, ^32^ Collectively, our data suggest that there is no benefit of early initiation of G‐CSF for hematopoietic recovery regardless of graft type.

It has been reported that G‐CSF could stimulate AML cells.^14^ Therefore, the risk of relapse in AML has been a concern regarding G‐CSF administration following HCT. However, our data showed that administration of G‐CSF did not increase relapse of AML following BMT, PBSCT, or CBT, irrespective of cytogenetic risk or disease status at HCT. Therefore, administration of G‐CSF can be considered after allogeneic HCT for AML despite poor cytogenetic risk or non‐CR status at HCT. On the other hand, most previous studies have shown that administration of G‐CSF did not improve survival despite its acceleration of neutrophil recovery.^1^, ^2^, ^3^, ^4^, ^5^, ^6^, ^8^, ^9^, ^10^, ^11^, ^13^ In our study, administration of G‐CSF and timing of initiation had almost no effect on NRM following BMT, PBSCT, or CBT, but administration of G‐CSF significantly improved OS and LFS only following CBT. Regarding the optimal timing to initiate G‐CSF for OS, late initiation of G‐CSF was the best timing for improved OS and LFS following CBT in the multivariate analysis. Therefore, our data suggest that G‐CSF should be routinely administered following CBT in adult patients with AML.

We had several limitations in this study. First, this was a registry‐based retrospective study in Japan, and it is unclear whether planned G‐CSF was administered or not. Although a meta‐analysis demonstrated that G‐CSF reduced the risk of documented infection and duration of parenteral antibiotics,^33^ we were unable to evaluate these results. Second, data on types of G‐CSF (e.g., filgrastim or lenograstim) and route and dose of G‐CSF used were insufficient in our registry data, which could affect outcomes after allogeneic HCT. Third, cost‐effectiveness could be considered for the use and timing of G‐CSF initiation after allogeneic HCT,^4^, ^5^, ^13^ but this could not be evaluated in our study.

In summary, our data show that compared to no administration of G‐CSF, administration of G‐CSF significantly accelerated neutrophil recovery after BMT, PBSCT, and CBT but was significantly associated with a higher risk of grades II–IV acute GVHD, irrespective of graft type. Additionally, an increased incidence of overall chronic GVHD with G‐CSF administration was observed in BMT and CBT patients, but not PBSCT patients. Finally, administration of G‐CSF significantly improved OS and LFS only following CBT. Based on these data, G‐CSF should be routinely administered for CBT in adult patients with AML. Regarding the timing to initiate G‐CSF, there is no benefit of early initiation for hematopoietic recovery regardless of graft type. The optimal timing of G‐CSF initiation remains unclear and needs further study.

## AUTHOR CONTRIBUTIONS

TK designed the research, analyzed the data, performed the statistical analysis, and wrote the first draft of the manuscript. MY contributed to the critical review of the manuscript. All the other authors contributed to data collection. All authors approved the final version.

## CONFLICT OF INTEREST STATEMENT

The authors declare no competing interests.

## PATIENT CONSENT STATEMENT

Written informed consent was obtained before registration for the Transplant Registry Unified Management Program (TRUMP).

## Supporting information


**Figure S1.** The effect of G‐CSF administration on grades III and IV acute GVHD (A–C), extensive chronic GVHD (D–F), and platelet recovery (G–I) according to graft type.

**Figure S2.** The effect of G‐CSF administration on relapse (A–C), non‐relapse mortality (D–F), and leukemia‐free survival (G–I) according to graft type.

**Figure S3.** Forest plots for the adjusted hazard ratios (HR) and 95% confidence intervals (CI) of G‐CSF administration of neutrophil recovery (A), relapse (B), and overall mortality (1‐OS) (C) among each graft type in subgroup analysis.

**Figure S4.** The effect of administration and timing to start with G‐CSF on grades III and IV acute GVHD (A–C), extensive chronic GVHD (D–F), and platelet recovery (G–I) according to graft type.

**Figure S5.** The effect of administration and timing to start with G‐CSF on relapse (A–C), non‐relapse mortality (D–F), and leukemia‐free survival (G–I) according to graft type.

**Figure S6.** The effect of administrationand timing to start with G‐CSF on 6‐months non‐relapse mortality (A–C), and 1‐year non‐relapse mortality (D–F) according to graft type.

**Figure S7.** The effect of administration and timing of G‐CSF initiation on posttransplant outcomes in haploidentical transplantation.

**Table S1.** Multivariate analysis of transplant outcomes of G‐CSF administration according to graft type.

**Table S2.** Multivariate analysis of transplant outcomes based on the administration and timing of G‐CSF initiation according to graft type.

**Table S3.** Multivariate analysis of 6 months‐ and 1 year non‐relapse mortality based on the timing of G‐CSF initiation according to graft type.

## Data Availability

The data of this study are not publicly available due to ethical restrictions that it exceeds the scope of the recipient/donor's consent for research use in the registry. Data may be available from the corresponding author upon reasonable request and with permission of the JSTCT/JDCHCT.
