# Supplementary material for: Different impacts of granulocyte colony‐stimulating factor administration on allogeneic hematopoietic cell transplant outcomes for adult acute myeloid leukemia according to graft type
Source: Am J Hematol. 2024 Nov 20;100(1):66–77. doi: 10.1002/ajh.27521 (PMC11625993; doi:10.1002/ajh.27521)
Supplement: Supplementary file 1 — Figure S1. The effect of G‐CSF administration on grades III and IV acute GVHD (A–C), extensive chronic GVHD (D–F), and platelet recovery (G–I) according to graft type. [file AJH-100-66-s003.pdf]

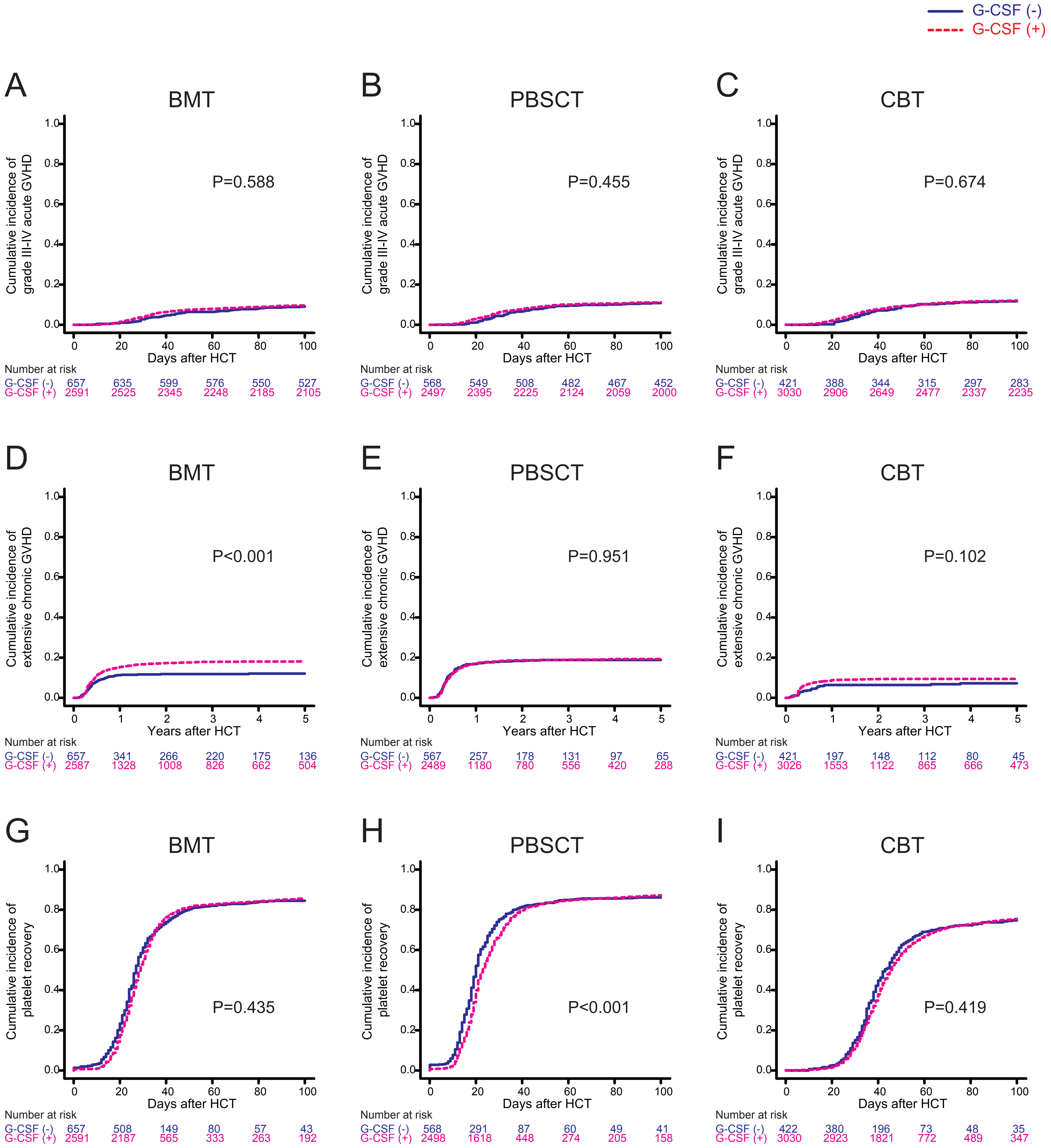

Supplementary Figure 1. The effect of G-CSF administration on grade III to IV acute GVHD (A-C), extensive chronic GVHD (D-F), and platelet recovery (G-I) according to graft type.
