# Supplementary material for: Different impacts of granulocyte colony‐stimulating factor administration on allogeneic hematopoietic cell transplant outcomes for adult acute myeloid leukemia according to graft type
Source: Am J Hematol. 2024 Nov 20;100(1):66–77. doi: 10.1002/ajh.27521 (PMC11625993; doi:10.1002/ajh.27521)
Supplement: Supplementary file 2 — Figure S2. The effect of G‐CSF administration on relapse (A–C), non‐relapse mortality (D–F), and leukemia‐free survival (G–I) according to graft type. [file AJH-100-66-s002.pdf]

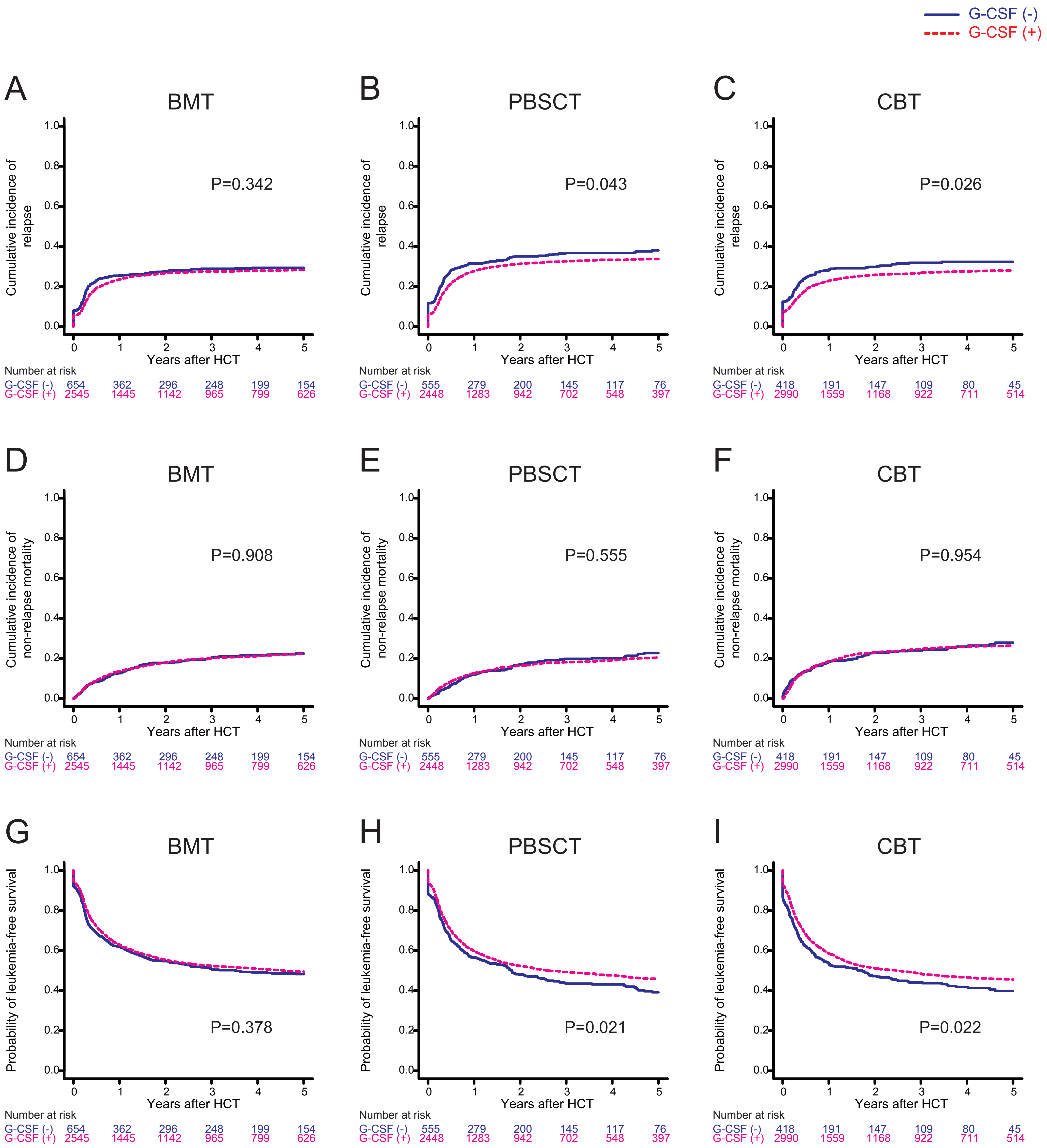

Supplementary Figure 2. The effect of G-CSF administration on relapse (A-C), non-relapse mortality (D-F), and leukemia-free survival (G-I) according to graft type.
