# Supplementary material for: Different impacts of granulocyte colony‐stimulating factor administration on allogeneic hematopoietic cell transplant outcomes for adult acute myeloid leukemia according to graft type
Source: Am J Hematol. 2024 Nov 20;100(1):66–77. doi: 10.1002/ajh.27521 (PMC11625993; doi:10.1002/ajh.27521)
Supplement: Supplementary file 3 — Figure S3. Forest plots for the adjusted hazard ratios (HR) and 95% confidence intervals (CI) of G‐CSF administration of neutrophil recovery (A), relapse (B), and overall mortality (1‐OS) (C) among each graft type in subgroup analysis. [file AJH-100-66-s001.pdf]

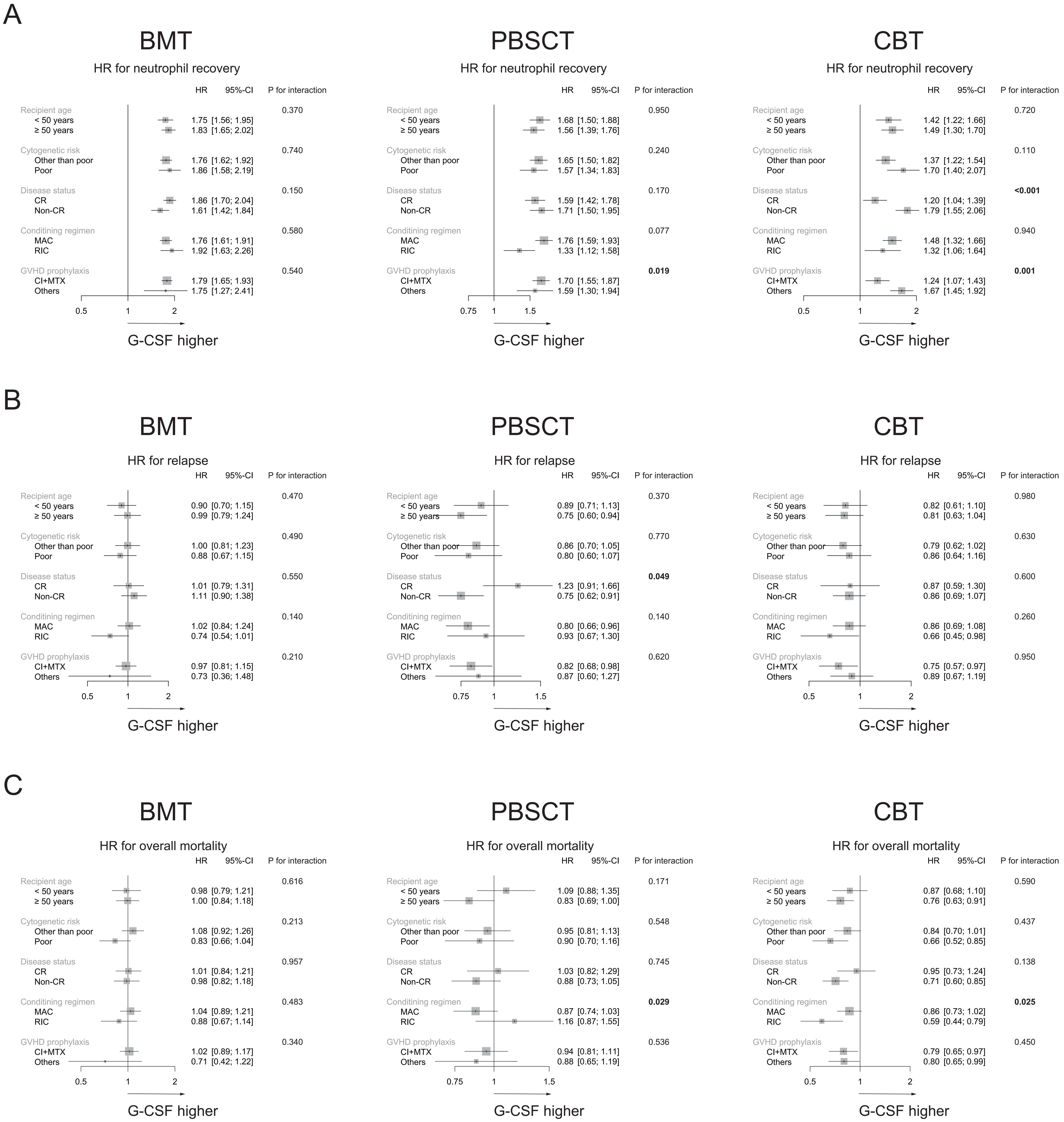

Supplementary Figure 3. Forest plots for the adjusted hazard ratios (HR) and 95% confidence intervals (CI) of G-CSF administration of neutrophil recovery (A), relapse (B), and overall mortality (1-OS) (C) among each graft type in subgroup analysis.
