# Supplementary material for: Different impacts of granulocyte colony‐stimulating factor administration on allogeneic hematopoietic cell transplant outcomes for adult acute myeloid leukemia according to graft type
Source: Am J Hematol. 2024 Nov 20;100(1):66–77. doi: 10.1002/ajh.27521 (PMC11625993; doi:10.1002/ajh.27521)
Supplement: Supplementary file 4 — Figure S4. The effect of administration and timing to start with G‐CSF on grades III and IV acute GVHD (A–C), extensive chronic GVHD (D–F), and platelet recovery (G–I) according to graft type. [file AJH-100-66-s005.pdf]

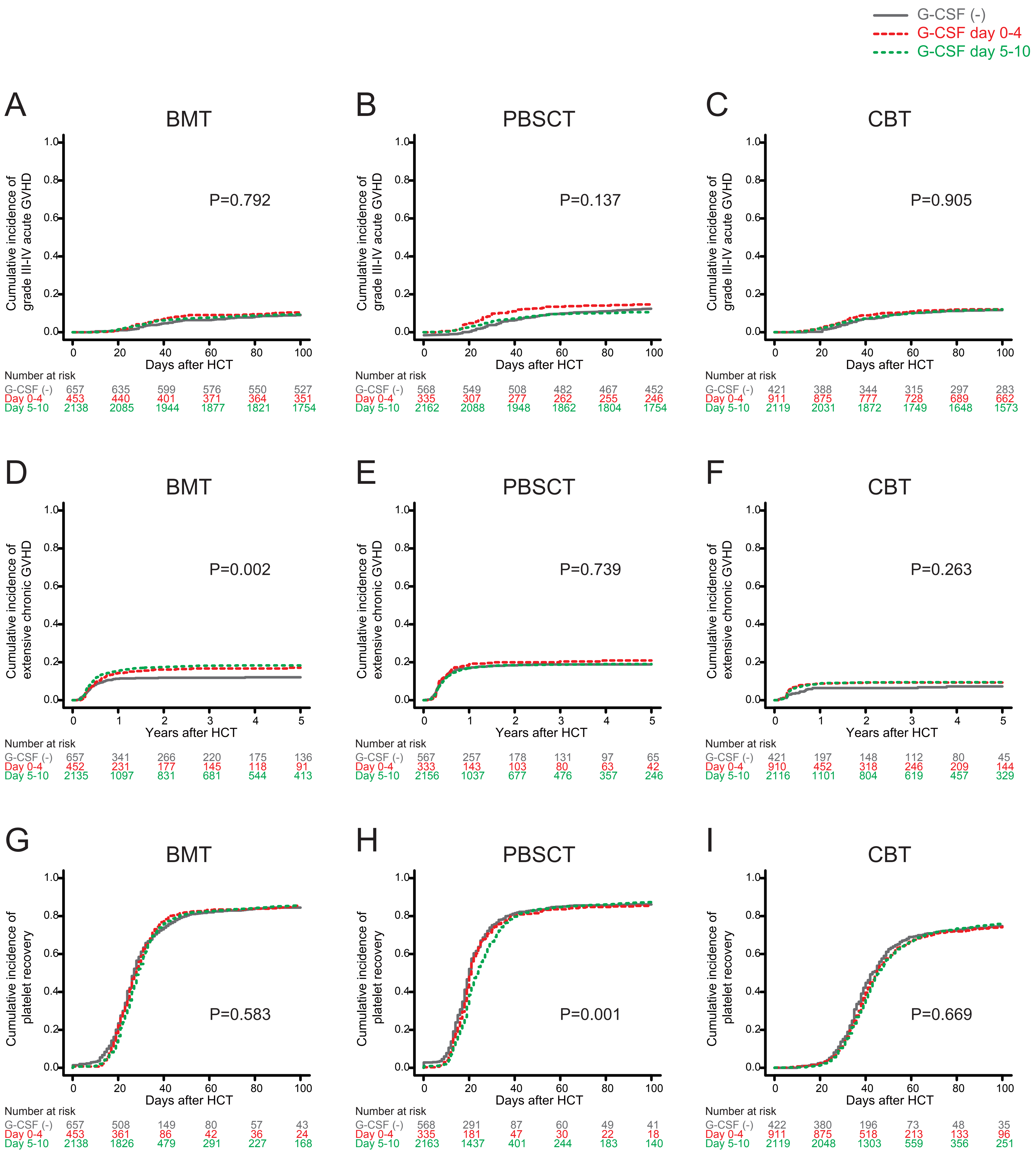

Supplementary Figure 4. The effect of administration and timing to start with G-CSF on grade III to IV acute GVHD (A-C), extensive chronic GVHD (D-F), and platelet recovery (G-I) according to graft type.
