# Supplementary material for: Different impacts of granulocyte colony‐stimulating factor administration on allogeneic hematopoietic cell transplant outcomes for adult acute myeloid leukemia according to graft type
Source: Am J Hematol. 2024 Nov 20;100(1):66–77. doi: 10.1002/ajh.27521 (PMC11625993; doi:10.1002/ajh.27521)
Supplement: Supplementary file 6 — Figure S6. The effect of administrationand timing to start with G‐CSF on 6‐months non‐relapse mortality (A–C), and 1‐year non‐relapse mortality (D–F) according to graft type. [file AJH-100-66-s007.pdf]

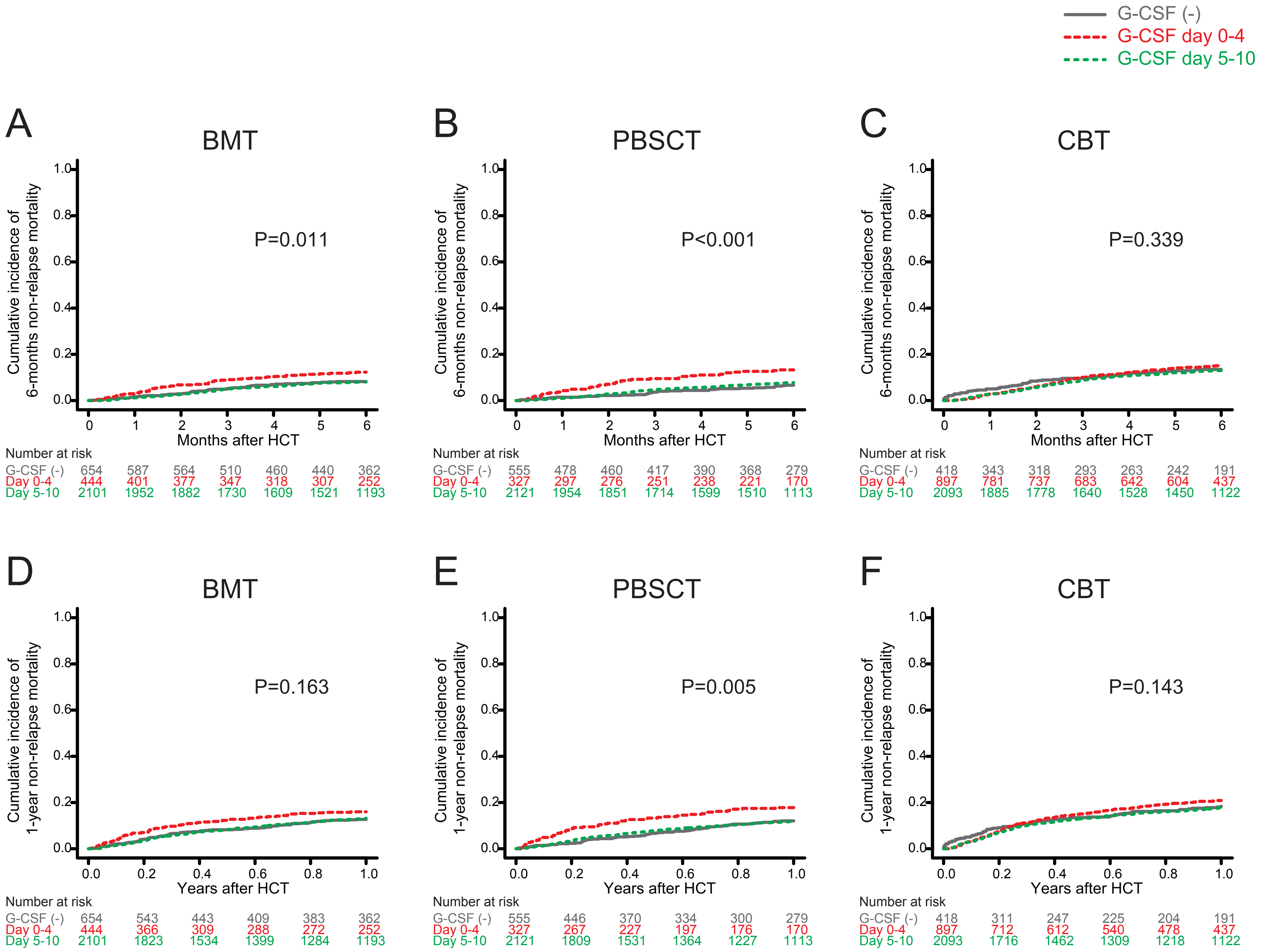

Supplementary Figure 6. The effect of administrationand timing to start with G-CSF on 6-months non-relapse mortality (A-C), and 1-year non-relapse mortality (D-F) according to graft type.
