# Supplementary material for: Different impacts of granulocyte colony‐stimulating factor administration on allogeneic hematopoietic cell transplant outcomes for adult acute myeloid leukemia according to graft type
Source: Am J Hematol. 2024 Nov 20;100(1):66–77. doi: 10.1002/ajh.27521 (PMC11625993; doi:10.1002/ajh.27521)
Supplement: Supplementary file 7 — Figure S7. The effect of administration and timing of G‐CSF initiation on posttransplant outcomes in haploidentical transplantation. [file AJH-100-66-s009.pdf]

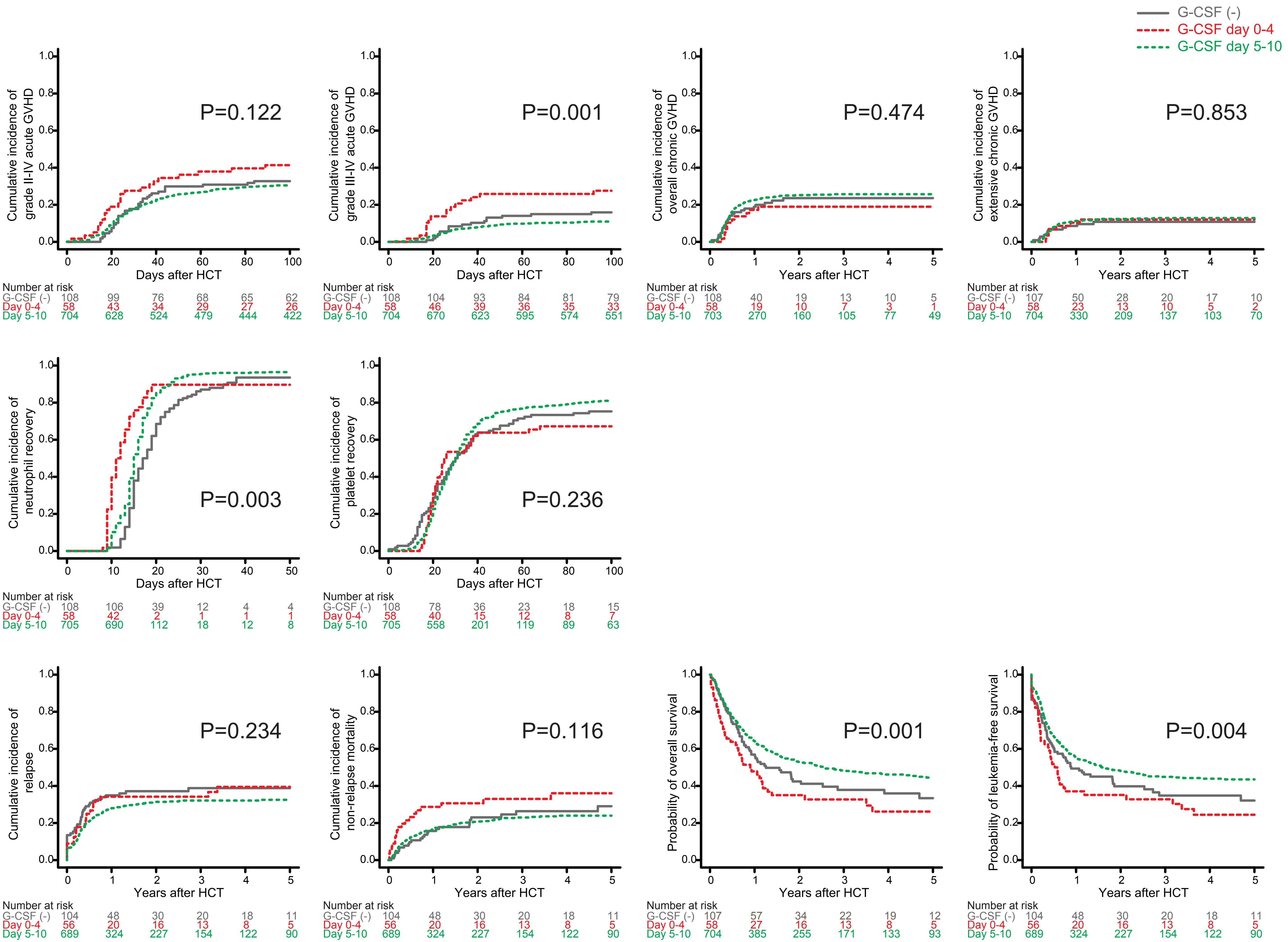

Supplementary Figure 7. The effect of administration and timing of G-CSF initiation on posttransplant outcomes in haploidentical transplantation.
