# Supplementary material for: Different impacts of granulocyte colony‐stimulating factor administration on allogeneic hematopoietic cell transplant outcomes for adult acute myeloid leukemia according to graft type
Source: Am J Hematol. 2024 Nov 20;100(1):66–77. doi: 10.1002/ajh.27521 (PMC11625993; doi:10.1002/ajh.27521)
Supplement: Supplementary file 8 — Table S1. Multivariate analysis of transplant outcomes of G‐CSF administration according to graft type. [file AJH-100-66-s004.docx]

**Supplementary Table 1**. Multivariate analysis of transplant outcomes of G-CSF administration according to graft type.

|  | BMT |  | PBSCT |  | CBT |  |
| --- | --- | --- | --- | --- | --- | --- |
|  | Adjusted HR (95%CI) | P | Adjusted HR (95%CI) | P | Adjusted HR (95%CI) | P |
| Grade II to IV acute GVHD | 1.24 (1.06-1.44) | **0.005** | 1.27 (1.07-1.52) | **0.006** | 1.21 (1.01-1.43) | **0.030** |
| Grade III to IV acute GVHD | 1.17 (0.88-1.56) | 0.260 | 1.20 (0.89-1.61) | 0.210 | 1.09 (0.80-1.48) | 0.570 |
| Overall chronic GVHD | 1.20 (1.00-1.43) | **0.040** | 0.94 (0.79-1.11) | 0.490 | 1.42 (1.10-1.84) | **0.007** |
| Extensive chronic GVHD | 1.46 (1.13-1.87) | **0.002** | 1.08 (0.86-1.34) | 0.490 | 1.37 (0.92-2.04) | 0.120 |
| Neutrophil recovery | 1.77 (1.64-1.91) | **<0.001** | 1.63 (1.50-1.77) | **<0.001** | 1.44 (1.30-1.60) | **<0.001** |
| Platelet recovery | 0.89 (0.80-0.98) | **0.019** | 0.83 (0.74-0.92) | **0.001** | 0.90 (0.80-1.02) | 0.120 |
| Relapse | 1.05 (0.88-1.25) | 0.530 | 0.88 (0.74-1.04) | 0.150 | 0.85 (0.70-1.03) | 0.110 |
| Non-relapse mortality | 1.01 (0.83-1.23) | 0.880 | 0.93 (0.75-1.17) | 0.590 | 0.96 (0.77-1.19) | 0.750 |
| Overall mortality (1-OS) | 0.99 (0.87-1.13) | 0.922 | 0.93 (0.81-1.07) | 0.335 | 0.79 (0.68-0.91) | **0.001** |
| Treatment failure (1-LFS) | 1.03 (0.91-1.17) | 0.616 | 0.88 (0.77-1.01) | 0.082 | 0.82 (0.71-0.95) | **0.008** |

GVHD, graft-versus-host disease; OS, overall survival; LFS, leukemia-free survival; BMT, bone marrow transplantation; PBSCT, peripheral blood stem cell transplantation; CBT, cord blood transplantation; HR, hazard ratio; CI, confidence interval.

The P-values in bold are statistically significant (<0.05).
