# Supplementary material for: Different impacts of granulocyte colony‐stimulating factor administration on allogeneic hematopoietic cell transplant outcomes for adult acute myeloid leukemia according to graft type
Source: Am J Hematol. 2024 Nov 20;100(1):66–77. doi: 10.1002/ajh.27521 (PMC11625993; doi:10.1002/ajh.27521)
Supplement: Supplementary file 9 — Table S2. Multivariate analysis of transplant outcomes based on the administration and timing of G‐CSF initiation according to graft type. [file AJH-100-66-s006.docx]

**Supplementary Table 2**. Multivariate analysis of transplant outcomes based on the administration and timing of G-CSF initiation according to graft type.

|  | BMT |  | PBSCT |  | CBT |  |
| --- | --- | --- | --- | --- | --- | --- |
|  | Adjusted HR (95%CI) | P | Adjusted HR (95%CI) | P | Adjusted HR (95%CI) | P |
| Grade II to IV acute GVHD |  |  |  |  |  |  |
| Early administration of G-CSF vs. none | 1.10 (0.88-1.36) | 0.380 | 1.26 (0.98-1.64) | 0.068 | 1.33 (1.09-1.61) | **0.003** |
| Late administration of G-CSF vs. none | 1.27 (1.09-1.48) | **0.002** | 1.28 (1.07-1.53) | **0.006** | 1.16 (0.97-1.38) | 0.097 |
| Late vs. early administration of G-CSF | 1.15 (0.96-1.38) | 0.110 | 1.00 (0.81-1.24) | 0.940 | 0.87 (0.76-0.98) | **0.032** |
| Grade III to IV acute GVHD |  |  |  |  |  |  |
| Early administration of G-CSF vs. none | 1.19 (0.80-1.76) | 0.380 | 1.40 (0.94-2.08) | 0.094 | 1.12 (0.79-1.58) | 0.500 |
| Late administration of G-CSF vs. none | 1.17 (0.87-1.56) | 0.280 | 1.17 (0.86-1.57) | 0.300 | 1.08 (0.79-1.47) | 0.630 |
| Late vs. early administration of G-CSF | 0.98 (0.71-1.36) | 0.920 | 0.83 (0.60-1.14) | 0.260 | 0.96 (0.76-1.20) | 0.730 |
| Overall chronic GVHD |  |  |  |  |  |  |
| Early administration of G-CSF vs. none | 1.08 (0.85-1.38) | 0.510 | 1.05 (0.82-1.33) | 0.680 | 1.37 (1.03-1.83) | **0.028** |
| Late administration of G-CSF vs. none | 1.22 (1.02-1.47) | **0.025** | 0.92 (0.78-1.09) | 0.370 | 1.44 (1.11-1.87) | **0.006** |
| Late vs. early administration of G-CSF | 1.13 (0.93-1.37) | 0.220 | 0.88 (0.72-1.07) | 0.220 | 1.04 (0.88-1.24) | 0.590 |
| Extensive chronic GVHD |  |  |  |  |  |  |
| Early administration of G-CSF vs. none | 1.33 (0.96-1.85) | 0.082 | 1.13 (0.82-1.55) | 0.450 | 1.32 (0.85-2.05) | 0.210 |
| Late administration of G-CSF vs. none | 1.48 (1.15-1.91) | **0.002** | 1.07 (0.85-1.34) | 0.540 | 1.39 (0.92-2.09) | 0.110 |
| Late vs. early administration of G-CSF | 1.11 (0.86-1.43) | 0.410 | 0.94 (0.72-1.24) | 0.700 | 1.05 (0.81-1.37) | 0.700 |
| Neutrophil recovery |  |  |  |  |  |  |
| Early administration of G-CSF vs. none | 1.74 (1.54-1.97) | **<0.001** | 1.72 (1.47-2.02) | **<0.001** | 1.44 (1.27-1.62) | **<0.001** |
| Late administration of G-CSF vs. none | 1.78 (1.65-1.92) | **<0.001** | 1.61 (1.48-1.75) | **<0.001** | 1.44 (1.30-1.60) | **<0.001** |
| Late vs. early administration of G-CSF | 1.02 (0.91-1.14) | 0.700 | 0.93 (0.80-1.08) | 0.390 | 1.00 (0.92-1.09) | 0.910 |
| Platelet recovery |  |  |  |  |  |  |
| Early administration of G-CSF vs. none | 0.93 (0.81-1.06) | 0.320 | 0.91 (0.77-1.07) | 0.260 | 0.92 (0.80-1.06) | 0.250 |
| Late administration of G-CSF vs. none | 0.88 (0.80-0.97) | **0.013** | 0.81 (0.73-0.91) | **<0.001** | 0.90 (0.79-1.02) | 0.100 |
| Late vs. early administration of G-CSF | 0.94 (0.84-1.05) | 0.320 | 0.90 (0.78-1.02) | 0.120 | 0.97 (0.89-1.07) | 0.630 |
| Relapse |  |  |  |  |  |  |
| Early administration of G-CSF vs. none | 1.09 (0.85-1.40) | 0.470 | 0.69 (0.53-0.89) | **0.005** | 0.89 (0.71-1.12) | 0.350 |
| Late administration of G-CSF vs. none | 1.04 (0.87-1.25) | 0.590 | 0.91 (0.77-1.09) | 0.340 | 0.83 (0.68-1.02) | 0.080 |
| Late vs. early administration of G-CSF | 0.95 (0.77-1.18) | 0.680 | 1.32 (1.05-1.66) | **0.014** | 0.92 (0.79-1.08) | 0.360 |
| Non-relapse mortality |  |  |  |  |  |  |
| Early administration of G-CSF vs. none | 1.07 (0.82-1.41) | 0.580 | 1.30 (0.96-1.76) | 0.084 | 1.05 (0.82-1.33) | 0.670 |
| Late administration of G-CSF vs. none | 1.00 (0.82-1.22) | 0.980 | 0.87 (0.69-1.10) | 0.260 | 0.92 (0.74-1.15) | 0.510 |
| Late vs. early administration of G-CSF | 0.93 (0.74-1.16) | 0.530 | 0.67 (0.52-0.86) | **0.002** | 0.88 (0.75-1.02) | 0.110 |
| Overall mortality (1-OS) |  |  |  |  |  |  |
| Early administration of G-CSF vs. none | 1.11 (0.92-1.33) | 0.254 | 1.03 (0.84-1.25) | 0.755 | 0.86 (0.73-1.02) | 0.092 |
| Late administration of G-CSF vs. none | 0.97 (0.84-1.11) | 0.670 | 0.91 (0.79-1.05) | 0.227 | 0.75 (0.65-0.88) | **<0.001** |
| Late vs. early administration of G-CSF | 0.87 (0.74-1.02) | 0.086 | 0.88 (0.75-1.05) | 0.164 | 0.87 (0.78-0.97) | **0.019** |
| Treatment failure (1-LFS) |  |  |  |  |  |  |
| Early administration of G-CSF vs. none | 1.29 (0.91-1.81) | 0.140 | 1.13 (0.94-1.35) | 0.172 | 0.90 (0.77-1.06) | 0.248 |
| Late administration of G-CSF vs. none | 1.06 (0.82-1.38) | 0.610 | 1.01 (0.89-1.15) | 0.827 | 0.79 (0.68-0.91) | **0.001** |
| Late vs. early administration of G-CSF | 0.89 (0.77-1.04) | 0.155 | 1.00 (0.85-1.18) | 0.980 | 0.86 (0.77-0.96) | **0.012** |

GVHD, graft-versus-host disease; OS, overall survival; LFS, leukemia-free survival; BMT, bone marrow transplantation; PBSCT, peripheral blood stem cell transplantation; CBT, cord blood transplantation; HR, hazard ratio; CI, confidence interval.

The P-values in bold are statistically significant (<0.05).
