# Supplementary material for: Different impacts of granulocyte colony‐stimulating factor administration on allogeneic hematopoietic cell transplant outcomes for adult acute myeloid leukemia according to graft type
Source: Am J Hematol. 2024 Nov 20;100(1):66–77. doi: 10.1002/ajh.27521 (PMC11625993; doi:10.1002/ajh.27521)
Supplement: Supplementary file 10 — Table S3. Multivariate analysis of 6 months‐ and 1 year non‐relapse mortality based on the timing of G‐CSF initiation according to graft type. [file AJH-100-66-s010.docx]

**Supplementary Table 3**. Multivariate analysis of 6 months- and 1 year- non-relapse mortality based on the timing of G-CSF initiation according to graft type.

|  | BMT |  | PBSCT |  | CBT |  |
| --- | --- | --- | --- | --- | --- | --- |
|  | Adjusted HR (95%CI) | P | Adjusted HR (95%CI) | P | Adjusted HR (95%CI) | P |
| 6 months- non-relapse mortality |  |  |  |  |  |  |
| Early administration of G-CSF vs. none | 1.53 (1.03-2.29) | **0.035** | 2.16 (1.32-3.53) | **0.001** | 1.07 (0.77-1.48) | 0.690 |
| Late administration of G-CSF vs. none | 1.04 (0.76-1.43) | 0.790 | 1.28 (0.86-1.90) | 0.210 | 0.94 (0.69-1.28) | 0.700 |
| Late vs. early administration of G-CSF | 0.67 (0.48-0.94) | **0.020** | 0.59 (0.41-0.86) | **0.005** | 0.88 (0.71-1.08) | 0.240 |
| 1 year- non-relapse mortality |  |  |  |  |  |  |
| Early administration of G-CSF vs. none | 1.29 (0.91-1.81) | 0.140 | 1.53 (1.04-2.26) | **0.029** | 1.08 (0.81-1.43) | 0.590 |
| Late administration of G-CSF vs. none | 1.06 (0.82-1.38) | 0.610 | 1.04 (0.77-1.39) | 0.780 | 0.93 (0.72-1.21) | 0.630 |
| Late vs. early administration of G-CSF | 0.82 (0.62-1.09) | 0.190 | 0.67 (0.49-0.92) | **0.016** | 0.86 (0.72-1.03) | 0.120 |

GVHD, graft-versus-host disease; OS, overall survival; LFS, leukemia-free survival; BMT, bone marrow transplantation; PBSCT, peripheral blood stem cell transplantation; CBT, cord blood transplantation; HR, hazard ratio; CI, confidence interval.

The P-values in bold are statistically significant (<0.05).
